# Infectious SARS-CoV-2 Is Emitted in Aerosol Particles

**DOI:** 10.1128/mBio.02527-21

**Published:** 2021-10-19

**Authors:** Seth A. Hawks, Aaron J. Prussin, Sarah C. Kuchinsky, Jin Pan, Linsey C. Marr, Nisha K. Duggal

**Affiliations:** a Department of Biomedical Sciences and Pathobiology, Virginia-Maryland College of Veterinary Medicine, Virginia Polytechnic Institute and State University, Blacksburg, Virginia, USA; b Department of Civil and Environmental Engineering, Virginia Polytechnic Institute and State University, Blacksburg, Virginia, USA; University of Florida

**Keywords:** animal models, coronavirus, transmission

## Abstract

Respiratory viruses such as SARS-CoV-2 are transmitted in respiratory droplets and aerosol particles, which are released during talking, breathing, coughing, and sneezing. Noncontact transmission of SARS-CoV-2 has been demonstrated, suggesting transmission via virus carried through the air. Here, we demonstrate that golden Syrian hamsters produce infectious SARS-CoV-2 in aerosol particles prior to and concurrent with the onset of mild clinical signs of disease. The average emission rate in this study was 25 infectious virions/hour on days 1 and 2 postinoculation, with average viral RNA levels 200-fold higher than infectious virus in aerosol particles. The majority of virus was contained within particles <5 μm in size. Thus, we provide direct evidence that, in hamsters, SARS-CoV-2 is an airborne virus.

## INTRODUCTION

SARS-CoV-2 is a respiratory virus that has caused more than 225 million cases and 4.6 million deaths as of September 2021 (https://covid19.who.int/). The virus is expelled from infected individuals in respiratory droplets that are produced during coughing, sneezing, talking, and breathing. The droplets can vary widely in size from less than 1 μm to about 1,000 μm, and the smaller ones may evaporate to less than half their initial diameter into a semisolid or solid particle (1, 2). Here, we refer to those smaller than 100 μm (3) as “aerosol particles” or simply “particles”; technically, an “aerosol” is a suspension of solid or liquid particles in a gas (4). Size is an important determinant of how droplets and particles travel through the air, so it significantly impacts transmission risk and mode. Those smaller than 10 μm remain suspended in air for many minutes to hours, during which they can travel long distances; this does not rule out their potential to transmit at close range, too (5, 6).

Infectious SARS-CoV-2 has been cultured from airborne particles sampled near COVID-19 patients (7, 8). SARS-CoV-2 has also been isolated from particles <1 μm within a car driven by a COVID-19 patient with mild illness (9). The collection of exhaled breath is a noninvasive sampling method that has been used to assess the airborne transmission potential of respiratory viruses, including seasonal human coronaviruses, influenza viruses, and rhinoviruses (10–13). For COVID-19 patients, SARS-CoV-2 RNA was detected in exhaled breath (14, 15). In nonhuman primates, SARS-CoV-2 RNA has also been detected in aerosol particles collected from inoculated animals (16, 17).

Hamsters are a naturally susceptible animal model for SARS-CoV-2 transmission and develop few clinical signs of disease (18–23). Importantly, inoculated hamsters have been shown to transmit SARS-CoV-2 to naive hamsters via noncontact transmission (18, 19, 23), suggesting SARS-CoV-2 may be transmitted between animals via aerosol particles released during breathing. However, infectious SARS-CoV-2 has not yet been successfully cultured from aerosol particles released from infected animals.

In this study, we sought to determine the shedding kinetics of infectious SAS-CoV-2 in aerosol particles. We found that hamsters inoculated with SARS-CoV-2 emit infectious SARS-CoV-2 into the air prior to and concurrent with the onset of mild clinical signs of disease. Viral titers decreased quickly over time, whereas viral RNA was detected in particles collected from the air for many days postinoculation. We also found that particles <5 μm contain the majority of airborne virus.

## RESULTS

### Particle generation by hamsters.

**FIG 1:**
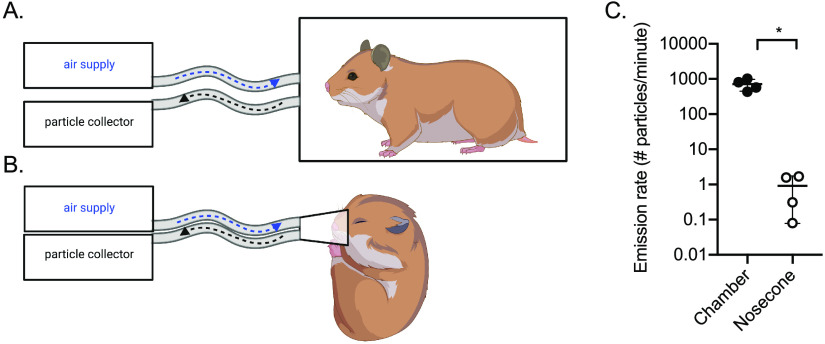
Air sampling methods. Aerosol particles were collected using a condensation sampler or an aerodynamic particle sizer. (A) Air was collected from awake hamsters within a sealed 2-liter chamber. (B) Air was collected from anesthetized hamsters using a nosecone. (C) Aerosol particle emission rate from uninfected hamsters (*n* = 4) in the chamber (filled circles) or by nosecone (open circles). *, *P* < 0.05. This figure was created with BioRender.com.

To test whether inoculated animals shed SARS-CoV-2 in aerosol particles, we established two sampling methods for hamsters. In the first method, animals were allowed to move freely within an empty 2-liter chamber (Fig. 1A). In the second method, animals were anesthetized, and a nosecone was placed over the nose and mouth (Fig. 1B). Attempts were made to restrain hamsters for the nosecone sampling without anesthesia but were unsuccessful. Particles were sampled via an outlet port from the chamber or nosecone.

We measured particles produced by uninfected hamsters and calculated the emission rate. Using the chamber, we found an average of 700 particles emitted per minute per hamster (Fig. 1C), with 99.9% of them <10 μm in size (Fig. S1 in the supplemental material). Using the nosecone, we found an average of 1 particle emitted per minute per hamster, which was significantly fewer than measured using the chamber approach (*P* < 0.05). The size distribution was similar in both cases, with very small particles being the most abundant.

10.1128/mBio.02527-21.1 FIG S1 Particle size distributions. Size distributions of aerosol particles generated from uninfected hamsters in the chamber (A) or through a nosecone (B). Each size distribution curve was obtained at times corresponding to the highest aerosol particle concentration during the 15-minute sampling period for each hamster. Download FIG S1, EPS file, 0.02 MB. Copyright © 2021 Hawks et al. 2021 Hawks et al. https://creativecommons.org/licenses/by/4.0/ This content is distributed under the terms of the Creative Commons Attribution 4.0 International license.

### Infectious SARS-CoV-2 is emitted in aerosol particles.

**FIG 2:**
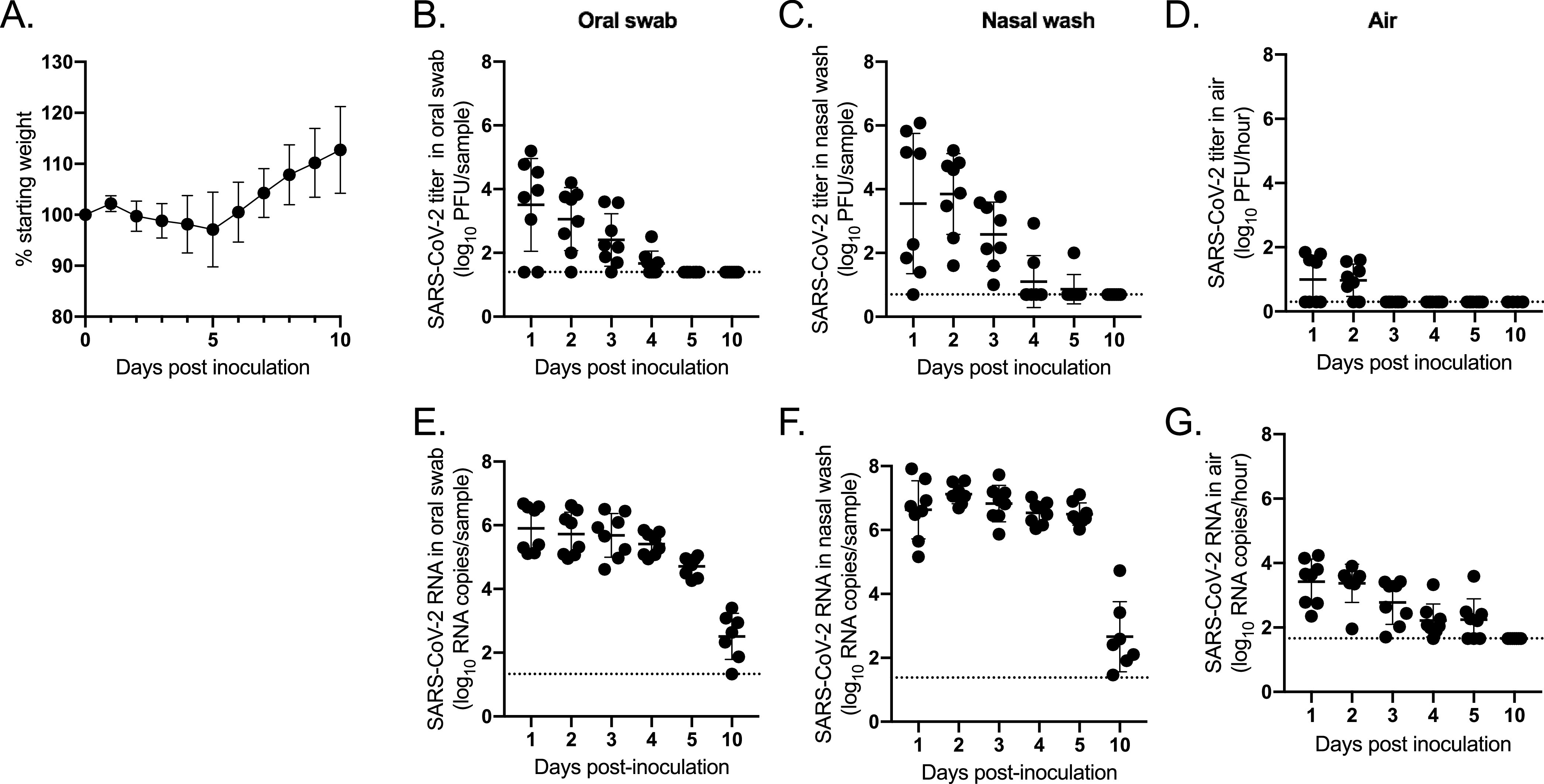
Emission of SARS-CoV-2 in aerosol particles. Hamsters (*n* = 8) were inoculated intranasally with SARS-CoV-2. (A) Percent starting weight; (B) viral titers in oral swabs; (C) viral titers in nasal washes; (D) viral titers in air samples; (E) viral RNA levels in oral swabs; (F) viral RNA levels in nasal washes; (G) viral RNA levels in air samples. Dashed line represents limit of detection.

In two independent experiments, hamsters were inoculated intranasally with SARS-CoV-2 strain USA-WA1/2020. Mild weight loss occurred 2 through 5 days postinoculation (dpi) (Fig. 2A). Oral swabs and nasal washes were collected daily. Virus peaked 1 dpi in the oral swabs at 3.5-log_10_ PFU/swab and 2 dpi in the nasal washes at 3.9-log_10_ PFU/wash (Fig. 2B and C). Significantly higher viral titers were observed in nasal washes from males than females 1 dpi, with a 5,000-fold difference (*P* < 0.001) (Fig. S2C). Viral titers for females peaked 1 day later than males, which was consistent between independent experiments. Viral titers decreased over time, with infectious virus below the limit of detection by 5 dpi for most animals. Samples were tested for SARS-CoV-2 RNA, which was detectable through 10 dpi (Fig. 2E and F). Sex-specific differences were not observed for viral RNA levels (Fig. S2E and F).

10.1128/mBio.02527-21.2 FIG S2 Sex-specific differences in emission of SARS-CoV-2 in aerosol particles. Male (*n* = 4) and female (*n* = 4) hamsters were inoculated intranasally with SARS-CoV-2. (A) Percentage of starting weight; (B) viral titers in oral swabs; (C) viral titers in nasal washes; (D) viral titers in air samples; (E) viral RNA levels in oral swabs; (F) viral RNA levels in nasal washes; (G) viral RNA levels in air samples. Dashed line represents limit of detection. **, *P* < 0.01; ***, *P* < 0001. Download FIG S2, EPS file, 0.4 MB. Copyright © 2021 Hawks et al. 2021 Hawks et al. https://creativecommons.org/licenses/by/4.0/ This content is distributed under the terms of the Creative Commons Attribution 4.0 International license.

Air samples were collected daily using the chamber approach for 1 h with a condensation sampler, which maintains viral infectivity (7). Infectious virus was detected in particles collected 1 and 2 dpi, with a mean emission rate of 1.4-log_10_ PFU/hour (Fig. 2D). Significantly greater infectious viral titers were detected in air samples from males than females 1 dpi (*P* < 0.01) (Fig. S2D). A majority (75%) of inoculated animals released detectable levels of virus in the air 2 dpi, and emission rates ranged from 0.9- to 1.8-log_10_ PFU/hour. Infectious virus was below the limit of detection in samples collected after 2 dpi. Air samples were also tested for viral RNA; viral RNA was detected through 5 dpi, with levels below the limit of detection by 10 dpi (Fig. 2G). Sex-specific differences were not observed for viral RNA levels in the air (Fig. S2G). For samples with detectable infectious virus, the RNA levels were approximately 200-fold higher than PFU levels on 1 and 2 dpi. Together, these data show that infectious SARS-CoV-2 is emitted in aerosol particles early in infection, prior to and concurrent with the onset of mild clinical signs of disease.

### Particles <5 μm contain infectious SARS-CoV-2.

**FIG 3:**
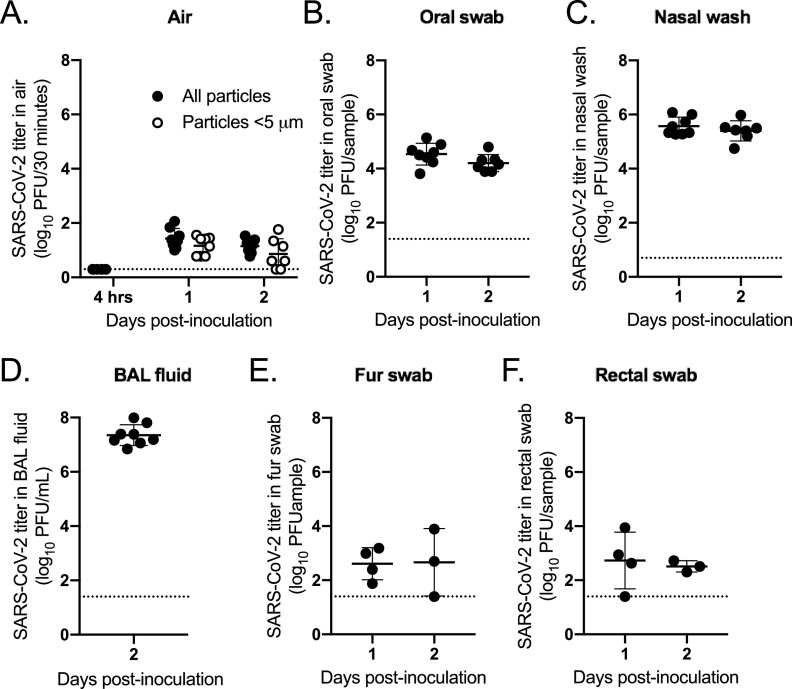
Aerosol particles <5 μm contain SARS-CoV-2. Hamsters (*n* = 8) were inoculated intranasally with SARS-CoV-2. (A) Viral titers in total particles (filled circles) or those <5 μm (open circles); (B) viral titers in oral swabs; (C) viral titers in nasal washes; (D) viral titers in BAL fluid; (E) viral titers in fur swabs; (F) viral titers in rectal swabs. Dashed line represents limit of detection.

Once we established the window of time postinoculation when infectious virus was detected in aerosol particles (1 to 2 dpi), we inoculated 8 additional hamsters in order to test whether small particles contain infectious SARS-CoV-2. Here, we shortened the air sampling time to 30 min. Male hamsters were used, as virus was more readily detected in their air samples compared to females’ air samples. To test whether the virus detected in the air was residual inoculum, we collected an air sample at 4 h postinoculation using the chamber; no virus was detected (Fig. 3A). Then, we collected air samples 1 and 2 dpi in the chamber with and without a cyclone separator, which removed particles >5 μm, placed upstream of the condensation sampler. We detected similar titers in samples collected with and without the cyclone separator, with no statistical significance in the difference in mean titers on 1 dpi (*P* = 0.34) or 2 dpi (*P* = 0.37), indicating that size restriction of particles did not alter the amount of virus detected (Fig. 3A). To test whether SARS-CoV-2 was detectable in the breath, we anesthetized the hamsters and collected their breath from the nosecone for 1 h. Infectious virus was not detectable. However, very few total particles were collected with this method compared to the chamber method (Fig. 1C).

High levels of virus were detectable in oral swabs, nasal washes, and bronchoalveolar lavage (BAL) fluid collected from the animals (Fig. 3B to D). A low level of virus was detected in fur and rectal swabs taken from the animals, which indicates that some of the airborne virus detected in the chamber could be resuspended from the body (Fig. 3E and F). Together, these results indicate that SARS-CoV-2 is emitted primarily in particles <5 μm.

## DISCUSSION

In this study, we found that hamsters emitted infectious SARS-CoV-2 in particles primarily <5 μm in size prior to or concurrent with mild clinical disease, with an emission rate of 1.4-log_10_ PFU/hour (Fig. 2 and 3). SARS-CoV-2 viral RNA was detected in aerosol particles and the upper respiratory tract for a longer duration than infectious virus. The upper respiratory tract (nasal wash) and lower respiratory tract (BAL fluid and lung tissue) contained high titers of virus, suggesting that the virus isolated from the air was primarily derived from the breath. However, we found that the fur was contaminated with low levels of infectious virus, indicating that virus resuspended from the fur may also be a mechanism of SARS-CoV-2 emission into the air.

Particles <5 μm in size can remain airborne and be inhaled. Thus, our results suggest that airborne transmission is likely a major driver of SARS-CoV-2 transmission. Our studies support reports of infectious SARS-CoV-2 collected in the air near COVID-19 patients (7, 9) and in their exhaled breath (24), as well as studies showing noncontact transmission of SARS-CoV-2 between ferrets and between hamsters (18, 19, 23), including one study that demonstrated transmission over a 1-m distance (25). One previous study has been unable to culture virus in exhaled breath from COVID-19 patients (26), which could be due to unknown or late collection times postonset of disease. Another study was unable to culture virus from aerosol particles collected from nonhuman primates (17), possibly due to reliance on equipment and buffers that do not maintain viral infectivity. Our results support the idea that transmission is likely to occur prior to or concurrent with symptom onset or in the absence of severe clinical disease, supporting studies that have found that asymptomatic infection is a major driver of community transmission (27, 28). Identifying the modes of SARS-CoV-2 transmission is critical to designing interventions to effectively prevent transmission, and our results support the use of masks and ventilation to reduce SARS-CoV-2 transmission.

This study was limited by our inability to collect respiratory particles directly from the hamsters. We were unable to detect infectious virus directly from the breath when anesthetized; however, anesthesia decreases the breath rate of hamsters (29), and we detected 700-fold fewer aerosol particles with this method (Fig. 1). Thus, given the detection of infectious virus on the fur, we cannot exclude that virus resuspended from the fur during movement may have contributed to the particles that we detected using the chamber method. Movement in guinea pigs has been shown to increase influenza particles in the air by increasing the resuspension of dust particles from the body (30). The contribution of resuspended particles to SARS-CoV-2 airborne transmission has not been studied.

In this study, we detected a delay in infectious virus emission in female hamsters compared to male hamsters. Sex-specific differences in COVID-19 disease severity are widely reported, with more severe disease in men (31; https://globalhealth5050.org/the-sex-gender-and-covid-19-project). It is unclear whether these sex-specific differences in infectious viral titers in nasal washes and air samples are relevant to human transmission. Lower cellular levels of a factor required for infection, such as ACE2 (32), may lead to lower viral emission in female hamsters. Interestingly, viral RNA levels in nasal washes and air samples were not significantly different between male and female hamsters. Thus, a factor that alters immune responses and is more highly expressed in females, such as estrogen (33), may enhance the release of defective virions. If the sex difference is only observed with infectious virus, rather than viral RNA, many studies may miss sex differences in viral titers, leading to the underestimation of differences in viral transmission potential between sexes. Sex-specific differences in infectious viral titers have not been observed by other groups, but differences in timing of samples collected may be relevant, as the largest difference was observed on 1 dpi in this study, which was not tested in other studies (34, 35).

Public health agencies have recently begun describing SARS-CoV-2 as an airborne virus. Here, we show that infectious virus is indeed culturable from the air early after infection, with the majority of particles containing infectious virus <5 μm in size. This suggests that SARS-CoV-2 may be maintained in the air for hours and over larger distances than previously recognized, with ventilation being an important tool for preventing transmission. Future studies will be critical for establishing the transmission potential of aerosol particles containing SARS-CoV-2.

## MATERIALS AND METHODS

### Ethics statement.

All animal experiments were approved by the Institutional Biosafety Committee and Institutional Animal Care and Use Committee at Virginia Polytechnic Institute and State University (IACUC protocol 20-184).

### Viruses and cell lines.

Inoculations were performed using SARS-CoV-2 strain USA-WA1/2020 (BEI Resources, NIAID, NIH; NR-52281), which was passaged once in Vero E6 cells followed by once in Vero cells upon receipt. Briefly, cells at approximately 80% confluence were inoculated with SARS-CoV-2, and supernatant was harvested when cytopathic effect (CPE) was present. Cells were cultivated in Dulbecco’s modified Eagle medium (DMEM) (Corning; product no. 10-017-CV) containing 5% gamma-irradiated fetal bovine serum (FBS) (VWR; catalog no. 97068-086), 100 units/ml penicillin, and 100 μg/ml streptomycin (Gibco; catalog no. 15140122) and maintained at 37°C and 5% CO_2_. All experiments involving infectious SARS-CoV-2 were performed in biosafety level 3 (BSL3) containment, which includes class II A2 biosafety cabinets (BSC) and powered air-purifying respirators.

### Animal studies.

All animal studies were performed in two independent experiments, with all work performed in AAALAC-accredited animal BSL3 (ABSL3) facilities under an approved IACUC protocol. The hamsters (Mesocricetus auratus) were sourced from an approved commercial vendor, Envigo (Indianapolis, IN). Animals had health reports that indicated there was no evidence of any underlying pathogens. Hamsters were housed in biocontainment caging on individually ventilated caging (IVC) racks manufactured by Allentown, Inc. and were provided food and water *ad libitum.* Hamsters received Teklad diet formulation 2918, a natural ingredient, irradiated feed (6% fat, 18% protein; Envigo). The caging contained Teklad diamond dry cellulose bedding (Envigo). Cages were changed weekly by transferring animals to clean cages. Hamsters were housed at a cage density of 2 hamsters per cage. The light cycle was 12 h of light/12 h of dark. Hamsters were monitored daily during the day. Hamsters were acclimated to the environment for 3 days prior to the study.

All manipulations were performed within a BSC. Animals were transported to the BSC within individual cages for all manipulations. Three- to 6-week-old golden Syrian hamsters were inoculated intranasally via both nostrils with 10^5^ PFU of SARS-CoV-2 in 100 μl Dulbecco phosphate-buffered saline (DPBS) (pH 7.4; Genesee Scientific). Hamsters were weighed and monitored for clinical signs of illness daily, including weight loss, lethargy, ruffled fur, or labored breathing. The mean weight for male hamsters was 68.3 ± 8.9 g, and the mean weight for female hamsters was 70.8 ± 4.9 g. Air samples, oral swabs, nasal washes, fur swabs, and rectal swabs were collected from each hamster daily. Nasal washes were collected by directly pipetting 50 μl viral transport media (1× M199 plus Hanks’ salts [Sigma; catalog no. M9163], 0.005 M Tris-HCl, 1% [wt/vol] bovine serum albumin, 2 mM l-glutamine, 0.35g/liter sodium bicarbonate, 100 U/ml penicillin, 100 μg/ml streptomycin, and 1 μg/ml amphotericin B) into each nostril. Swab samples were collected by prewetting sterile flocked swabs (Puritan Medical Products; catalog no. 25-3306-H) in 500 μl viral transport media, placing the swab inside the cavity, making 2 to 4 circular passes against the mucosal surfaces, immersing the swab in the viral transport media, and then freezing. Swabs and nasal washes were collected after air sampling while under isoflurane or ketamine/xylazine anesthesia, depending on whether the chamber or nosecone method was used (see “Particle sampling and measurement”). Hamsters were anesthetized by inhalation of 3% isoflurane or by intraperitoneal (i.p.) injection with a mixture of ketamine (100 mg/kg) and xylazine (10 mg/kg) until the animal was nonresponsive to a toe pinch. After sample collection, hamsters were monitored for recovery from anesthesia (resuming normal movement). All samples were frozen at −80°C prior to testing. At the termination of studies, or when clinical signs of disease, such as 15% weight loss, were present, hamsters were euthanized via CO_2_ inhalation. Posteuthanasia, bronchoalveolar lavage (BAL) fluid was collected as previously described (36); whole lungs were also collected. Relative humidity and temperature in the animal facility during the studies were 27.1% ± 11% and 24.6 ± 0.8°C, respectively.

### Particle sampling and measurement.

**(i) Chamber and nosecone.** Particles generated by hamsters were sampled using two different approaches. In the first, infected hamsters were placed in a 2-liter (3.75 in. by 9.00 in. by 3.75 in.) sealed chamber (VetEquip) in which they were allowed to move freely. Clean air (Airgas; part no. OX USP300) was supplied from a PerkinElmer gas anesthesia system through an inlet to the chamber at a flow rate of 1.0 or 1.5 liter/min to match the flow rate of the equipment used to sample particles through an outlet (see section below). This approach captured total particles produced in exhaled breath, released from the fur, and resuspended from the chamber floor by the hamster’s activity. The second approach captured particles produced in exhaled breath only. Infected hamsters were anesthetized with 100 mg/kg ketamine and 10 mg/kg xylazine. After the hamster was fully immobile, a nonrebreather nosecone (Kent Scientific; VetFlo-0802) was placed on the hamster. Air was provided through the nosecone’s inlet tube, and particles were sampled via the outlet port.

**(ii) Infectious virus collection.** A water-based condensation particle sampler (Aerosol Devices Inc.; series 110A) was connected to the outlet of the chamber or nosecone. Superior to traditional methods for collecting infectious virus (37), this technique has been used to detect infectious SARS-CoV-2 in a hospital and car and directly in exhaled breath of patients (7, 9). The sampler was operated at 1.5 liter/min for a total of 1 h, collecting particles directly into a vial containing 400 μl viral transport medium. To separate particles by size, an air sampling cyclone device (cyclone) (URG-2000-30E-5-2.5-S; URG, Chapel Hill, NC) that removes particles larger than 5 μm at the sampling flow rate used here was installed upstream of the condensation sampler. Samples were collected for 30 min with the cyclone and 30 min without the cyclone. For 4 hamsters, the cyclone was used during the first 30-minute period, and for 4 hamsters, the cyclone was used during the second 30-minute period.

**(iii) Particle size distribution.** An aerodynamic particle sizer (TSI; model 3321) was connected to the outlet of the chamber or nosecone to measure the particle size distribution for 15 min in 1-min intervals at a flow rate of 1 liter/min, with makeup air provided at the same flow rate. This instrument detects particles across the size range of 0.5 to 20 μm. The first 2 min were discarded to prevent carryover between measurements. Background concentrations were measured in an empty chamber and nosecone to estimate the contribution from the hamsters alone and were subtracted from aerosol measurements.

### Virus quantification.

**(i) Plaque assays.** Infectious virus was quantified via Vero cell plaque assay. Briefly, samples were serially diluted 10-fold, plated onto confluent Vero cells in a 6-well plate, and incubated for 1 h at 37°C to allow for viral adsorption. After the 1-h adsorption period, 2 ml of an 0.8% (wt/vol) agarose overlay medium was added to the wells. Plates were incubated for 1 day at 37°C, after which a second 2-ml overlay containing 3% neutral red was added to the wells. One day later, plaques were counted. The limit of detection was 5 PFU/ml for air samples, which were tested neat, and 50 PFU/ml for all other samples, which were tested at a 1:10 dilution. This was converted to “per sample” to reflect the entire sample, as the volumes differed between sample type as follows: 25 PFU/swab (oral/fur/rectal) and BAL fluid (500 μl), 2 PFU/air sample (400 μl), and 5 PFU/nasal wash (100 μl).

**(ii) Real-time RT-PCR.** SARS-CoV-2 RNA was extracted from samples via Qiagen QIAamp viral RNA minikit. RNA was quantified by real-time reverse transcriptase PCR (RT-PCR) using the 2019-nCoV RUO primer/probe kit (IDT; catalog no. 10006713) and Bio-Rad iTaq universal probes one-step kit. Synthetic SARS-CoV-2 RNA (BEI, NIAID, NIH; NR-52358) was used for a standard curve. The limit of detection was 1.33-log_10_ RNA copies/oral swab, 1.66-log_10_ RNA copies/air sample, and 1.38-log_10_ RNA copies/nasal wash.

### Statistics.

Samples were compared using a mixed-effects analysis with Sidak’s correction for multiple comparisons. All statistics were performed in GraphPad Prism 9.
